# Acute Pain and a Motivational Pathway in Adult Rats: Influence of Early Life Pain Experience

**DOI:** 10.1371/journal.pone.0034316

**Published:** 2012-03-28

**Authors:** Lucie A. Low, Maria Fitzgerald

**Affiliations:** 1 Division of Neuroscience, Physiology and Pharmacology, University College London, London, United Kingdom; 2 Alan Edwards Centre for Research on Pain, McGill University, Montreal, Quebec, Canada; Southern Illinois University School of Medicine, United States of America

## Abstract

**Background:**

The importance of neonatal experience upon behaviour in later life is increasingly recognised. The overlap between pain and reward pathways led us to hypothesise that neonatal pain experience influences reward-related pathways and behaviours in adulthood.

**Methodology/Principal Findings:**

Rat pups received repeat plantar skin incisions (neonatal IN) or control procedures (neonatal anesthesia only, AN) at postnatal days (P)3, 10 and 17. When adult, rats with neonatal ‘pain history’ showed greater sensory sensitivity than control rats following acute plantar skin incision. Motivational behaviour in the two groups of rats was tested in a novelty-induced hypophagia (NIH) paradigm. The sensitivity of this paradigm to pain-induced changes in motivational behaviour was shown by significant increases in the time spent in the central zone of the arena (43.7±5.9% vs. 22.5±6.7%, p<0.05), close to centrally placed food treats, and decreased number of rears (9.5±1.4 vs. 19.2±2.3, p<0.001) in rats with acute plantar skin incision compared to naive, uninjured animals. Rats with a neonatal ‘pain history’ showed the same pain-induced behaviour in the novelty-induced hypophagia paradigm as controls. However, differences were observed in reward-related neural activity between the two groups. Two hours after behavioural testing, brains were harvested and neuronal activity mapped using c-Fos expression in lateral hypothalamic orexin neurons, part of a specific reward seeking pathway. Pain-induced activity in orexin neurons of control rats (18.4±2.8%) was the same as in uninjured naive animals (15.5±2.6%), but in those rats with a ‘pain history’, orexinergic activity was significantly increased (27.2±4.1%, p<0.01). Furthermore the extent of orexin neuron activation in individual rats with a ‘pain history’ was highly correlated with their motivational behaviour (r = −0.86, p = 0.01).

**Conclusions/Significance:**

These results show that acute pain alters motivational behaviour and that neonatal pain experience causes long-term changes in brain motivational orexinergic pathways, known to modulate mesolimbic dopaminergic reward circuitry.

## Introduction

Studies on the long-term impact of early-life experience upon the developing nervous system have shown that infant experience, including pain, can have profound effects on adult neural processing [1]–[12]. Repeated nociceptive stimulation during human infancy causes changes in sensory and nociceptive processing lasting for many months or years after injury [13]–[18]. This is clinically important, as preterm neonates in intensive care may experience multiple painful procedures per day [19], [20], yet analgesia for these infants is poorly managed and understood [21]–[23].

At the spinal cord level, the neurobiological mechanisms associated with or driving the long-term effects of early pain include alterations in spinal nociceptive processing [18], [24], [25], shifts in nociceptive withdrawal reflexes [26]–[28] driven by activity-dependent changes in primary afferent nerves and dorsal horn circuits [5], [29], [30], and altered gene expression [7]. This has led to the concept of a ‘critical period’ for nociceptive development, during which noxious input can shape and define nociceptive behaviour for life [28], [31]. However, the effects of early pain on cognitive processing in the brain, which requires integration from many CNS areas [32]–[34], are not well understood.

The purpose of these experiments was to investigate the long-term effects of repeated painful experience during early life on reward pathways and behaviours in the adult rat. The reward pathways of the brain overlap extensively with those involved in nociception [35], and a number of studies have shown that pain and reward processing are intrinsically linked: induction of the placebo effect releases endogenous opioids and dopamine from classic reward-processing areas such as the nucleus accumbens [36]–[38], and human fMRI studies have shown that painful stimuli activate reward-related brain areas [39], [40]. Furthermore, patients suffering from chronic pain show deficits in reward processing [41]. This extensive overlap in pain and reward processing suggests reward processing as a likely candidate to reflect effects of early life pain.

Here we use two methods to study acute pain-related activity in reward pathways in rats, and the effect of early life pain experience upon that activity. The first is a behavioural measure of motivation, using the concept of novelty-induced hypophagia (NIH). Hypophagia refers to inhibition of feeding, so we used a paradigm where the anxiety of moving into the centre of an open field is balanced by the reward of a sweet food treat placed there [42]–[46]. This paradigm is novel to the pain field, but is useful for exploring interactions between anxiety and motivational behaviours, as it exploits the conflict that rodents must resolve between rewarding exploration/approach behaviours, and avoidance of aversive environments [47]. We included outcome measures that were informative for both types of behaviour in order to discriminate between the two. The second method involved direct neuroanatomical visualisation of reward circuitry activation, using orexin cell activity in the lateral hypothalamus (LH) as a neurobiological marker. Orexins (hypocretins) are small peptides synthesised in the hypothalamus, and are involved in maintenance of wake states, arousal, and feeding [48]–[51]. LH-specific orexinergic cells are also strongly implicated in reward processing and addiction [52], [53], mediate dopaminergic signalling [54]–[59] and are analgesic in a number of animal pain models [60]–[63].

As pain and reward are intrinsically linked, and because the development of the nociceptive system is critically influenced by early pain experience, we hypothesised that peripheral injury during neonatal life would cause alterations in reward-related behaviours and neural pathways in later life, caused by interference with normal pain processing development during the ‘critical period’. Since the greatest changes in pain behaviour following early pain experience are seen after a later painful insult [6], [15], [18], [28], [29], [64], we hypothesised that changes in reward processing would be most pronounced in the presence of an acute painful stimulus.

## Methods

### 1. Ethics statement

Hooded Lister rats (Charles River, Margate, UK) were used in all experiments. Animals were kept in strict accordance with the United Kingdom Animals (Scientific Procedures) Act 1986. The project licence permit number issued by the UK Home Office was PPL70/6895, which specifically approved this study. The personal licence permit number (LAL) issued by the UK Home Office for this study was PIL 70/19402, which specifically approved this study under project licence number PPL70/6895, in accordance with the UK Home Office Animals (Scientific Procedures) Act 1986. Ethical treatment of all animals was ensured, and all attempts to minimize suffering of animals were made. Animals were maintained on a 12 hour light-dark cycle, with food and water access ad libitum. Litters were bred on-site and ranged in size from 6 to 16 pups.

### 2. Experimental design

To investigate the effects of neonatal injury on acute pain and motivation, plantar skin incision surgery (referred to as ‘skin incision’, IN) was performed under anesthesia in neonatal rats of both sexes at postnatal day (P)3 and repeated at P10 and P17. Control animals were repeatedly anesthetized at the same time points (AN).

When rats were adult, a sub group of these animals underwent an acute skin incision, thus producing two further groups, IN+IN and AN+IN. The final four groups of animals are summarised in Table 1. The left hindpaw was injured in all conditions.

**Table 1:** Summary of experimental groups.

Group #	Early life pain experience	Adult Pain experience	Abbreviation
1	Plantar skin incision	No treatment	IN
2	Anesthesia only	No treatment	AN
3	Plantar skin incision	Plantar skin incision	IN+IN
4	Anesthesia only	Plantar skin incision	AN+IN

### 3. Neonatal hindpaw skin incision surgery

On surgery days, the entire litter was removed from the dam and placed onto a heating pad with some home-cage bedding until all surgeries/anesthesias had been performed. Pups were randomly assigned to treatment groups at P3. Blinding was performed by photographing pups' skin/fur markings immediately after each procedure against a backdrop stating treatment group, and these pictures referred to at the next treatment time point. Unblinding was performed once data from all mature adult animals had been tested and scored.

Pups were anesthetized with 5% isoflurane and maintained at 2–3% anesthesia in 5 L/min oxygen. The left hindpaw of all animals was cleaned with iodine solution in order to standardise the smell of pups being returned to the dam, and help prevent the skin incised (IN) treatment group receiving more maternal attention to the injured hindpaw than the controls (anesthetized only – AN). Surgery/control anesthesia was repeated 3 times, first performed at postnatal day (P)3, and repeated at both P10 and P17. An incision was made with an 11 gauge scalpel into the plantar surface of the left hindpaw, from the midpoint of the heel to the proximal border of the first footpad as previously described [28], [65]. The underlying muscle and fascia were lifted and separated from surrounding tissue using bent needle-tipped forceps, then released and the skin sutured by 2 stitches with 5/0 Mersilk (Ethicon, UK). The wound was cleaned with iodine solution and animals were photographed and returned to the heated recovery container.

Anesthetized controls were induced in the same manner as injured animals and received the same duration of anesthesia, on the same days, as experimental animals. Control animals also had paws cleaned with iodine to limit olfactory differences between treatment groups. After surgery, litters were kept together until recovery of all pups, and the entire litter returned to the dam. After the final treatment, animals were weaned at P22-23, housed in standard conditions with same-sex littermates in groups of 2–3, and allowed to mature with minimal interference to adulthood (at least P90) before testing.

### 4. Acute adult hindpaw skin incision surgery

To create the final two experimental groups, when animals had reached adulthood (>P90), a single acute skin incision was carried out in the same manner as described above. Thus, two groups of adults with acute skin incision were created, one that had been repeatedly anesthetized as neonates (AN+IN) and one that had received repeated incision as neonates (IN+IN). Anaesthetic duration for all animals, in all groups, and at all ages, lasted 5–10 minutes.

### 5. Sensory testing

Sensory hypersensitivity was quantified both before and after acute injury in adulthood to a) confirm that neonatal injury affected pain hypersensitivity in our adult animals, in line with previous literature [6], [27], [28], [31], [58], [59], [89], and b) to establish an appropriate time point after acute injury, when animals were still hypersensitive, for behavioural assessment in the NIH arena.

Sensory testing was performed in AN and IN groups when animals were mature (P>90), to measure baseline responses prior to adult incision, and up to 5 days after acute adult surgery. Animals were habituated to testing apparatus for 30 minutes on 3 days prior to testing, and for 15 minutes on test days. Thermal and mechanical testing was counterbalanced. Animals tested for sensory sensitivity were not tested in the NIH arena.

Cutaneous mechanical sensitivity of the hindpaw was measured using von Frey hairs (vFh). Animals were placed into clear Plexiglas boxes elevated on wire mesh and von Frey hairs of increasing force applied to the plantar surface of the left (ipsilateral) hindpaw, immediately proximal to the incision site. Six applications of each hair were performed, with an inter-stimulus interval of at least 10 seconds between each application. Testing began with hair number 10 (1.95 g), and increased until threshold was reached – defined as the force at which the animal performed a paw withdrawal to 3 out of the 6 (50%) stimuli. The right (contralateral) paw was then tested in the same manner after an interval of at least 5 minutes.

To assess thermal sensitivity, animals were placed onto a clear glass sheet and a radiant heat source (55°C) focussed onto the plantar surface of the paw immediately proximal to the incision, and latency to withdrawal reflex measured automatically [66]. To prevent tissue damage, the test was terminated after 20 seconds if no withdrawal occurred. Three measurements were taken from each paw, with an inter-stimulus interval of at least 2 minutes between beam application, and the mean average withdrawal latency calculated.

### 6. Novelty-induced hyponeophagia (NIH) testing

Behavioural testing was only carried out in male rats. To familiarise animals to the palatable food treats (Cheerios® breakfast cereal, Nestlé), all animals received 5–10 Cheerios treats, placed onto the surface of their home-cage, for 3 days prior to testing. All animals readily sought and consumed treats in their home cage before testing in the NIH paradigm. All testing was performed between 10am and 12pm by a blinded experimenter in a quiet, brightly lit room.

**Figure 1:**
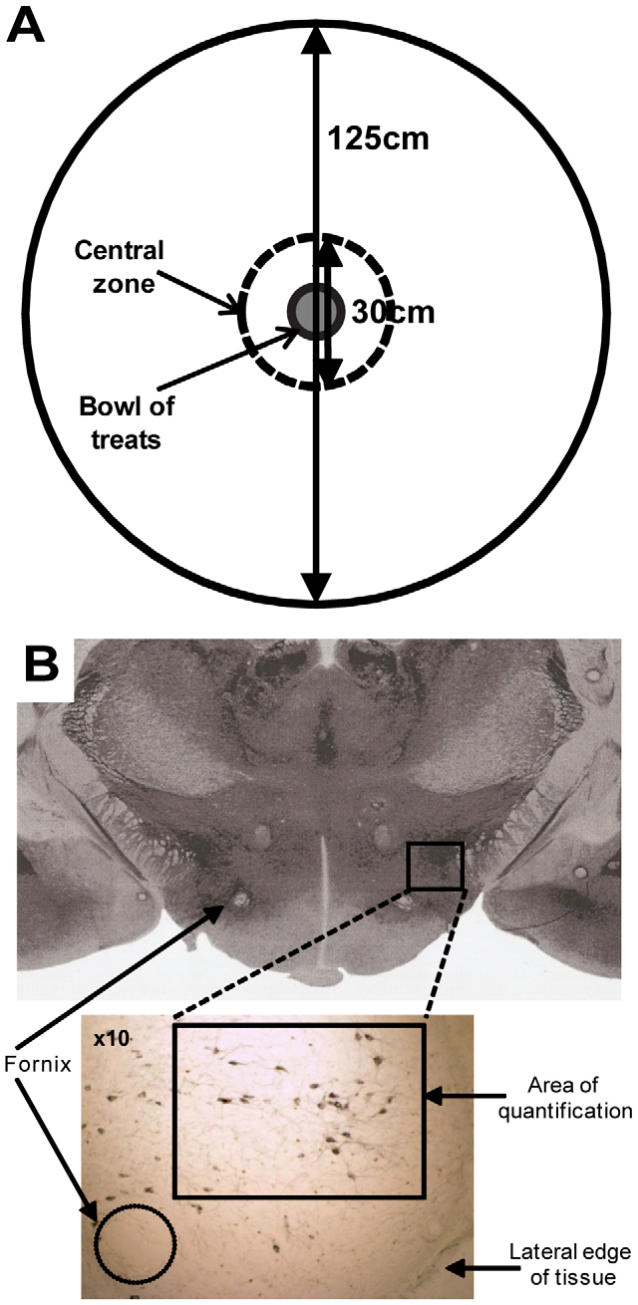
The novelty-induced hypophagia (NIH) arena, and localisation of the lateral hypothalamus. (**a**) At 125 cm in diameter with high (50 cm) walls, and painted white, the NIH arena is large, bright and anxiogenic. A bowl of 20 Cheerios® (Nestlé) food treats was placed in the central zone (CZ), defined as a circular area of diameter 30 cm. (**b**) The lateral hypothalamus (LH) was defined as lateral of the fornix, and bilaterally counted. Orexin cells are stained in black in the bottom panel. Top image taken from Paxinos and Watson (2004).

On the day of testing, animals were removed singly from their home cage and placed into a holding box with clean sawdust. The holding box was taken into the testing room and the animal immediately placed into the testing arena equidistant from the centre and the arena walls, with the experimenter then leaving the room. The NIH arena consisted of a circular, bright (>1000 lux) white open field 1.25 m in diameter with a wall height of 50 cm to prevent escape. The central zone (CZ) was defined as a circular area with 15 cm radius around the central point of the arena (Figure 1A).

Twenty treats were available in the centre of the arena in a glass bowl. To investigate the effects of presence or absence of food treats, separate subsets of animals in each experimental group were tested with or without food treats in the food bowl. Behaviour was recorded by camera (Logitech Quickcam, Logitech® Inc.) suspended above the arena for a period of 15 minutes with no human presence. After testing, the experimenter returned and animals were removed from the arena and placed back into the holding box, where a photograph was taken of their markings for future unblinding. The holding box was returned to the room containing the home cages.

Tested animals were kept separate from cage-mates awaiting testing, and so were retained in the holding box whilst the arena was cleaned in preparation for the next animal. Once both/all cage-mates had been tested, animals were returned to their home-cages. All animals were tested in the NIH arena once only. Sawdust in the holding box was changed after each cage of animals had been tested. After each test, the arena was scrubbed and disinfected with 5% Trigene and then 70% ethanol in preparation for the following animal, to remove any olfactory traces. Remaining treats were disposed of after each animal was tested, and the bowl washed with hot water and disinfected with 70% ethanol.

Videos of behaviour in the NIH area were manually scored by a blinded experimenter on the following outcome measures: number of faecal boli and urine puddles >5 mm diameter, number of rears, central zone entries, percentage of time spent in the central zone, time to eat first treat, and number of treats eaten.

### 7. Immunohistochemistry

2 hours after behavioural testing, animals were terminally anesthetized via i.p. overdose of pentobarbital, and transcardially perfused with 4% paraformaldehyde (PFA). Brains were removed, post-fixed in 4% PFA for four hours, then transferred to 30% sucrose and azide solution and stored at 4°C. For immunohistochemistry, sections (40 µm) were cut through the lateral hypothalamus (from Bregma: AP: −3–5 mm, DV: −7–9 mm, ML: +1.5–3 mm; and lateral of the fornix – Figure 1b, [67]) and blocked in 5% horse serum plus hydrogen peroxide for 1 hour. C-Fos primary antibody (1∶10,000; raised in rabbit; Calbiochem, Nottingham, UK) then secondary antibody (biotinylated anti-rabbit, raised in goat, 1∶500; Vector Laboratories Inc., CA, USA) were applied and the signal amplified using the Vectastain ABC kit (Vector Laboratories Inc., CA, USA). Brown DAB (3,3′-diaminobenzidine) staining (Vector Laboratories Inc., CA, USA) was used to visualise c-Fos protein in the nuclei of lateral hypothalamic cells. Next, sections were incubated with orexin-A primary antibody (1∶1000; raised in goat; Santa Cruz Biotechnology Inc., CA, USA) then a biotinylated anti-goat secondary antibody, raised in horse (1∶500; Vector Laboratories Inc., CA, USA) and the signal amplified using the Vectastain ABC kit as above. Blue DAB staining was performed to visualise orexin protein in the cytoplasm of lateral hypothalamic cells (Vector Laboratories Inc., CA, USA).

Cell counting was done by a blinded experimenter under light microscopy (Nikon E800). Orexin-positive cells, c-Fos-positive nuclei, and cells co-expressing orexin and c-Fos lateral of the fornix were counted (see Fig. 1B). Five sections were counted bilaterally per animal, with 5–8 animals per group (totalling between 50 and 80 sections counted per group). The percentage of orexin-positive cells co-expressing c-Fos was calculated. Averages were calculated within animal, then within group.

### 8. Data analysis

Data from both sexes were pooled in the sensory testing analysis to increase the sample size. Behavioural experiments in the NIH arena were only performed in males. For data analysis, two-way ANOVAs of all four treatment groups were performed for each outcome measure, with neonatal treatment and adult incision as variables. Number of rears, CZ entries, CZ time (%), and treats eaten were divided into 5 minute time bins and the second (5–10 minute) time bin analysed. This was done to minimise any confounds of anxiety-like behaviours exhibited during the first 5 minutes and familiarity effects in the final 5 minutes - time course analysis showed significant effects of time on all outcome measures but clearest group differences in the second time bin (data not shown). All other outcome measures represent behaviour over the total recording time. Animals that did not eat any treats during the entire recording period were excluded from the analyses of ‘Time to 1^st^ treat’ and ‘Number eaten’ outcome measures only, but included in all other analyses. This was done as including them in the analysis i.e. ‘time to 1^st^ treat’ automatically assigned each animal a false value of either 0 s or 360 s, both of which caused false ceiling effects. Similarly, inclusion of a ‘number eaten’ of 0 treats for non-eating animals would also skew data unfairly, when the fact that animals did not eat was a useful outcome measure in its own right.

Immunohistochemical data analyses consisted of two-way ANOVA with neonatal treatment and adult incision as variables. Pearson's correlation co-efficient was used to correlate the percentage of orexin cells co-expressing c-Fos with the behavioural outcome measures that showed significant differences between treatment groups. Normality testing (D'Agostino and Pearson omnibus normality test) was performed for all data, and analysed parametrically as appropriate. Bonferroni's post-hoc tests were performed to find the direction of any differences. Data were analysed with Prism version 4.01 (GraphPad Software Inc.) and are presented as the mean ± SEM.

## Results

### 1. Baseline sensory withdrawal thresholds are not affected by early life pain experience

Baseline flexion withdrawal thresholds to mechanical stimulation of the plantar hindpaw were tested in two groups of adult animals - those that had received a repeated plantar skin incision as neonates (IN, n = 5), and controls that were anesthetized as neonates (AN, n = 5). In neonatally injured animals, the adult baseline injured paw thresholds were no different from those of the uninjured (contralateral) paw, nor from those of the pooled ipsilateral and contralateral paws of control neonatally anesthetized rats (IN injured: 37.9±4.0 g, IN uninjured: 29.4±4.4 g, AN: 29.3±4.6 g; p = 0.42; one-way ANOVA; data not shown). Baseline thermal withdrawal thresholds were also not different between injured and uninjured paws in the neonatally incised group (n = 6) and controls (n = 6) (IN injured: 6.5±1.0 s, IN uninjured: 5.4±0.6 s, AN: 5.6±0.4; p = 0.49, one-way ANOVA; data not shown).

### 2. Hypersensitivity evoked by acute plantar skin incision is enhanced by early life pain experience

**Figure 2:**
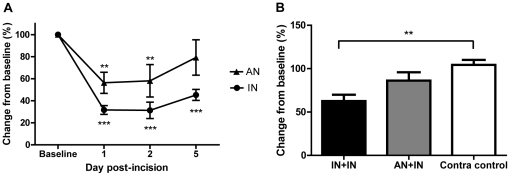
Acute skin incision causes drops in sensory thresholds that persist over 5 days. (**a**) Following the time course of plantar skin incision in the adult over five days post-surgery, both neonatally incised (IN) and control (AN) animals show a significant drop in mechanical withdrawal threshold in the injured paw compared to baseline (p<0.01 and p<0.001) at days 1 and 2 post-surgery. At day 5, only IN animals were still mechanically hypersensitive (p<0.001). IN animals were significantly more sensitive than AN animals (Treatment F_(1,24)_ = 6.59, p<0.05, 2-way RM ANOVA with Bonferroni post-tests). The contralateral paw showed no differences compared to baseline nor between paws (p>0.05, data not shown). (**b**) 2 days after adult incision, only neonatally incised (IN+IN, n = 6) animals still showed a decrease in thermal withdrawal threshold (i.e. thermal hypersensitivity) (p<0.01, One-way ANOVA with Bonferroni's Multiple Comparison Test). There was no significant percentage change in threshold in the injured paw of neonatally anesthetized (AN+IN, p>0.05, n = 6) animals 48 hours after an acute adult injury. **p<0.01, ***p<0.001.

After an acute adult skin incision, mechanical sensitivity to von Frey hair stimulation of the affected paw was followed over 5 days post-surgery. Figure 2A shows that both groups of acutely injured animals showed increased sensitivity in the injured paw (p<0.01) and that that the effect was greater in animals with a pain history - the mechanical hyperalgesia was greater in amplitude and longer in duration in the early life injured rats than in early anesthetized animals (2-way ANOVA, time and treatment as factors, Treatment F_(1,24)_ = 6.59, p<0.05; Time F_(3,24)_ = 21.51, p<0.0001).

Thermal sensitivity was also tested 48 hours post skin incision (Figure 2B). Thermal hyperalgesia was only observed in the injured paw of neonatally incised animals (IN+IN, n = 6). The injured paw of control, neonatally anesthetized animals (AN+IN, n = 6) showed no difference in sensitivity compared to the uninjured paws from both treatment groups (IN+IN: 3.8±0.6 s, AN+IN: 4.9±0.9 s. Contralateral uninjured (control): 5.7±0.5 s; p<0.05, one-way ANOVA).

These data show that hyperalgesia after a skin incision in adulthood is more pronounced in animals with a history of neonatal incision and that effects were still apparent at 48 hours after adult incision in both neonatally anesthetised and neonatally injured animals. This time point was therefore chosen for testing motivational behaviour in the novelty-induced hypophagia (NIH) paradigm.

### 3. Acute plantar skin incision in adults alters motivational behaviour

Behavioural experiments were undertaken in the novelty-induced hypophagia (NIH) arena on adult male rats from each group to establish the acute effects of plantar skin incision on motivational behaviour, and the influence of early life pain experience upon these behaviours. All behavioural testing was performed 48 hours after adult surgical treatment. Female data is not presented as hormonal fluctuations, which are known to affect behaviour, were not monitored.

**Figure 3:**
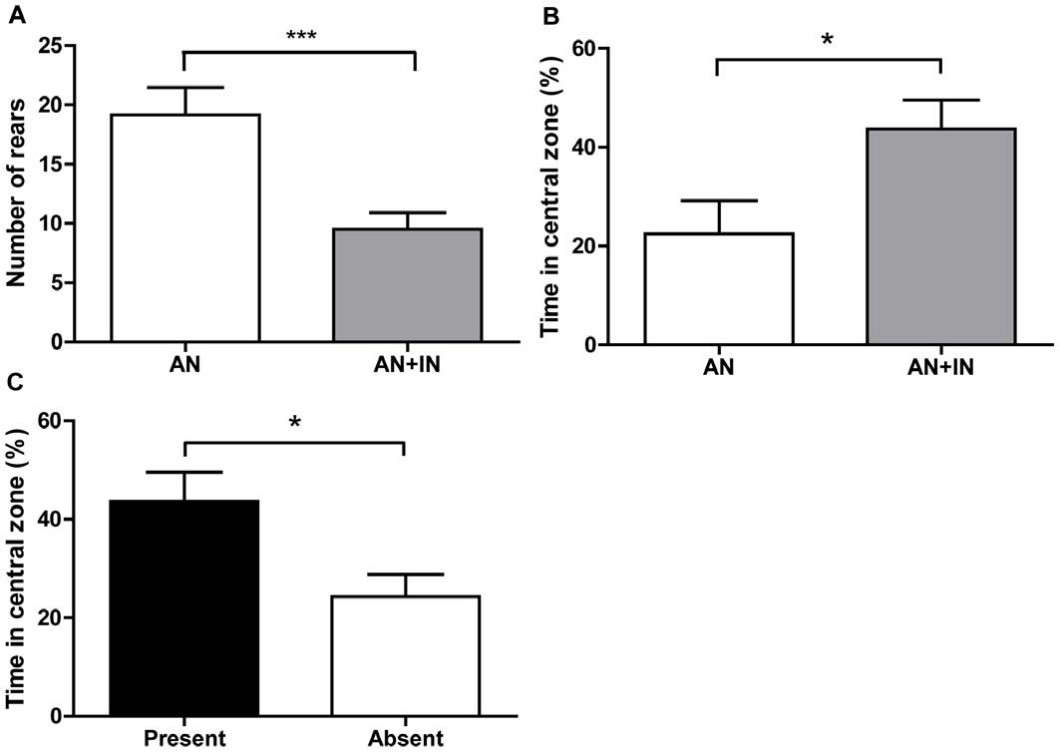
Acute skin incision affects motivational behaviour that is driven by presence of food treats. (**a**) Rearing behaviour decreased (F_(1,56)_ = 34.83; p<0.001) and (**b**) percentage of time spent in the central zone increased (F_(1,56)_ = 13.02); p<0.05) in the NIH arena when animals with no previous pain history were tested 48 hours after an acute adult injury. Graph illustrates AN+IN (n = 14) versus AN (n = 13) for clarity. (**c**) The increase in time spent in the CZ after acute incision is driven by the presence of treats - when treats were absent, adult incised animals spent less time in the central zone (p<0.05; all groups analysed via one-way ANOVA with Bonferroni's Multiple Comparison Test). *p<0.05, ***p<0.001.

To begin, the effect of an acute plantar incision to the hindpaw of adult rats was tested upon animals with no pain history. Two groups of neonatally anesthetized animals were compared, of which one had received a plantar incision 48 hours earlier (AN: n = 13, AN+IN: n = 14, Table 2). Figure 3 shows that animals receiving an acute plantar incision in adulthood (AN+IN) rear significantly less than non-injured controls in the NIH arena (Figure 3A, p<0.001) and spend significantly more time in the central zone (Figure 3B, p<0.05; two-way ANOVA), suggesting an increase in motivation to stay in the centre of the arena. Table 2 shows results for all outcome measures.

**Table 2:** NIH arena results for all four experimental groups.

Outcome Measure	Treatment Group			
	AN (n = 13)	IN (n = 19)	AN+IN (n = 14)	IN+IN (n = 14)
**Faeces**	2.15±0.55	1.95±0.35	3.36±0.70	**3.5±0.50** #
**Urine**	1.92±0.37	2.26±0.48	3.07±0.45	2.74±0.46
**Rears (5–10 mins)**	19.15±2.32	17.74±1.51	**9.50±1.41** ***	**8.14±0.17** ###
**CZ entries (5–10 mins)**	6.77±0.93	7.68±0.74	7.50±0.51	6.93±0.68
**CZ time (%)(5–10 mins)**	22.47±6.69	23.69±4.77	**43.69±5.89** *	**43.49±5.48** #
**Time to 1^st^ treat (s)**	479.0±51.54	374.5±26.53	378.5±24.48	416.5±46.90
**Number eaten (5–10 mins)**	5.0±1.17	6.42±0.73	8.1±0.66	7.21±0.91
**Non-eaters (p-value from expected 100% baseline)**	3/13 (0.22)	**7/19 (0.008)** ▴	2/14 (0.48)	2/14 (0.48)

AN = repeated neonatal anesthesia. IN = repeated neonatal skin incision. AN+IN = repeated neonatal anesthesia plus adult incision. IN+IN = repeated neonatal skin incision plus adult incision. Results are mean ± SEM. Significant results are highlighted in bold.

*p<0.05 and

***p<0.001, compared to AN group.

# p<0.05,

### p<0.001, compared to IN group; two-way ANOVA for each outcome measure with Bonferroni's Multiple Comparison Tests.

▴ compared to an expected baseline of 100% eaters; Fisher's Exact test.

Removing the treats from the central food-bowl confirmed that the presence of a food reward motivates movement into the central zone of the arena. Figure 3C shows that removal of food treats decreased the amount of time acutely injured animals spent in the central zone (AN+IN present (n = 14) vs. absent (n = 5), p<0.05, one-way ANOVA).

These experiments demonstrated that motivational behaviour, as measured by specific behaviour in the NIH arena, is sensitive to the presence of acute pain in adult rats and that behaviours are driven by the presence of treats.

### 4. Acute plantar skin incision in adults with a pain history also alters motivational behaviour

Next we wished to test the effect of early life pain experience upon acute pain-evoked motivational behaviours. In order to do this we first established whether baseline behaviours were influenced by early life experience. To test this we compared neonatally anesthetized and incised rats (AN or IN) in the NIH arena (Table 2). No significant differences between IN and AN treatment groups were found on any outcome measure, showing that early life injury *per se* does not affect NIH arena behaviour. However, of the 19 neonatally incised males tested, 7 did not eat any treats during the testing period; this was a significantly lower number than unexpected, as all animals readily consumed treats when presented with them in their home cage (Fishers exact test, comparing number of non-eaters against an expected eating baseline of 100%, p = 0.008, Table 2).

**Figure 4:**
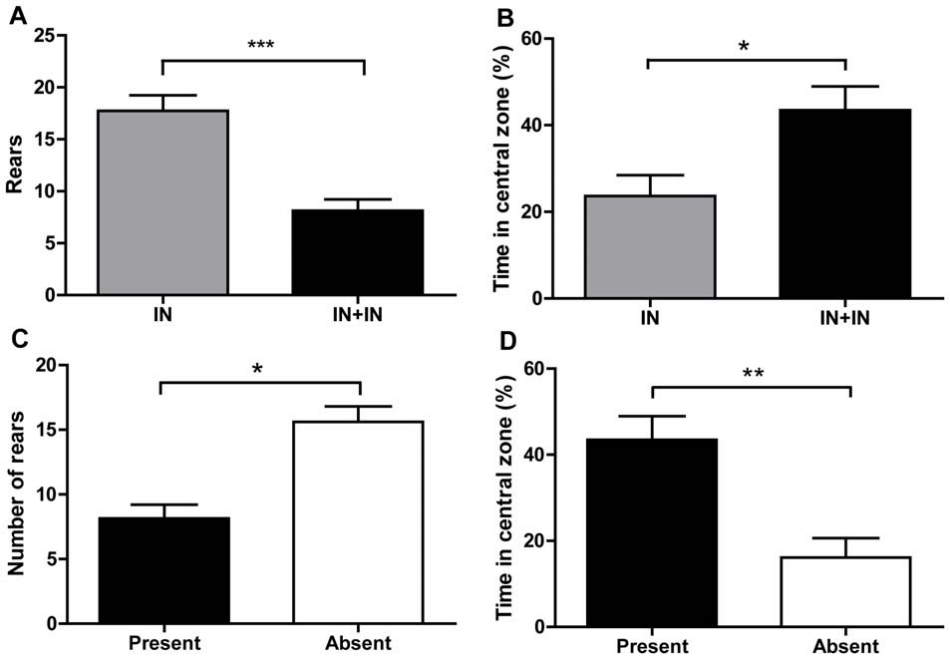
Re-incision to neonatally-injured animals affects motivational behaviours that are driven by presence of food treats. (**a**) Rearing behaviour decreased (F_(1,56)_ = 34.83; p<0.001) and (**b**) percentage of time spent in the central zone increased (F_(1,56)_ = 13.02); p<0.05) in neonatally incised animals with an adult re-incision (IN+IN, n = 14) versus neonatally incised alone (IN, n = 19). (**c**) The presence of treats affected both rearing and time spent in the central zone. When treats were absent (n = 5), animals reared more frequently (p<0.05) and (**d**) spent significantly less time in the central zone (p<0.01; all groups analysed via one-way ANOVA with Bonferroni's Multiple Comparison Test). *p<0.05, **p<0.01, ***p<0.001.

Next we tested whether animals with early life pain experience showed a change in motivational behaviour following an acute incision as adults. Figure 4 and Table 2 show that when rats with a pain history undergo skin incision as an adult, motivational behaviours are increased. Re-incised animals (IN+IN, n = 14) rear less frequently than those injured as neonates only (Figure 4A, IN, n = 19; p<0.001), spend more time in the central zone of the arena (Figure 4B, p<0.05), and produce more faecal boli, an index of anxiety (Table 2, p<0.05; all one-way ANOVA). Removing the treats from the central food-bowl again showed that these behaviours are driven by the presence of the treats. Figures 4C and D show that when treats were absent (n = 5), animals reared more frequently than when the food treats are present (p<0.05) and spent significantly less time in the central zone (p<0.01, one-way ANOVA).

Finally we tested whether the acute incision-evoked alterations in motivational behaviour are different in animals with or without early life pain experience. To test this, motivational behaviours were compared between acutely incised adult rats with a ‘pain history’ (IN+IN, n = 14) and acutely incised adult rats without one (AN+IN, n = 14). Table 2 shows that there is no significant difference on any outcome measure between these two treatment groups (p>0.05, 2-way ANOVA).

Overall, there are three key results from these behavioural data (i) neonatal pain experience does not affect baseline motivational behaviour of the adult rat in the NIH arena (ii) an acute incision in adulthood, whether it is an acute injury or a re-injury to a neonatally injured animal, increases the approach behaviours towards a food reward (iii) there is no significant difference in this acute pain-induced motivational behaviour in animals with or without a pain history.

### 5. Plantar skin incision activates the lateral hypothalamic orexin system in animals with early life pain experience

**Figure 5:**
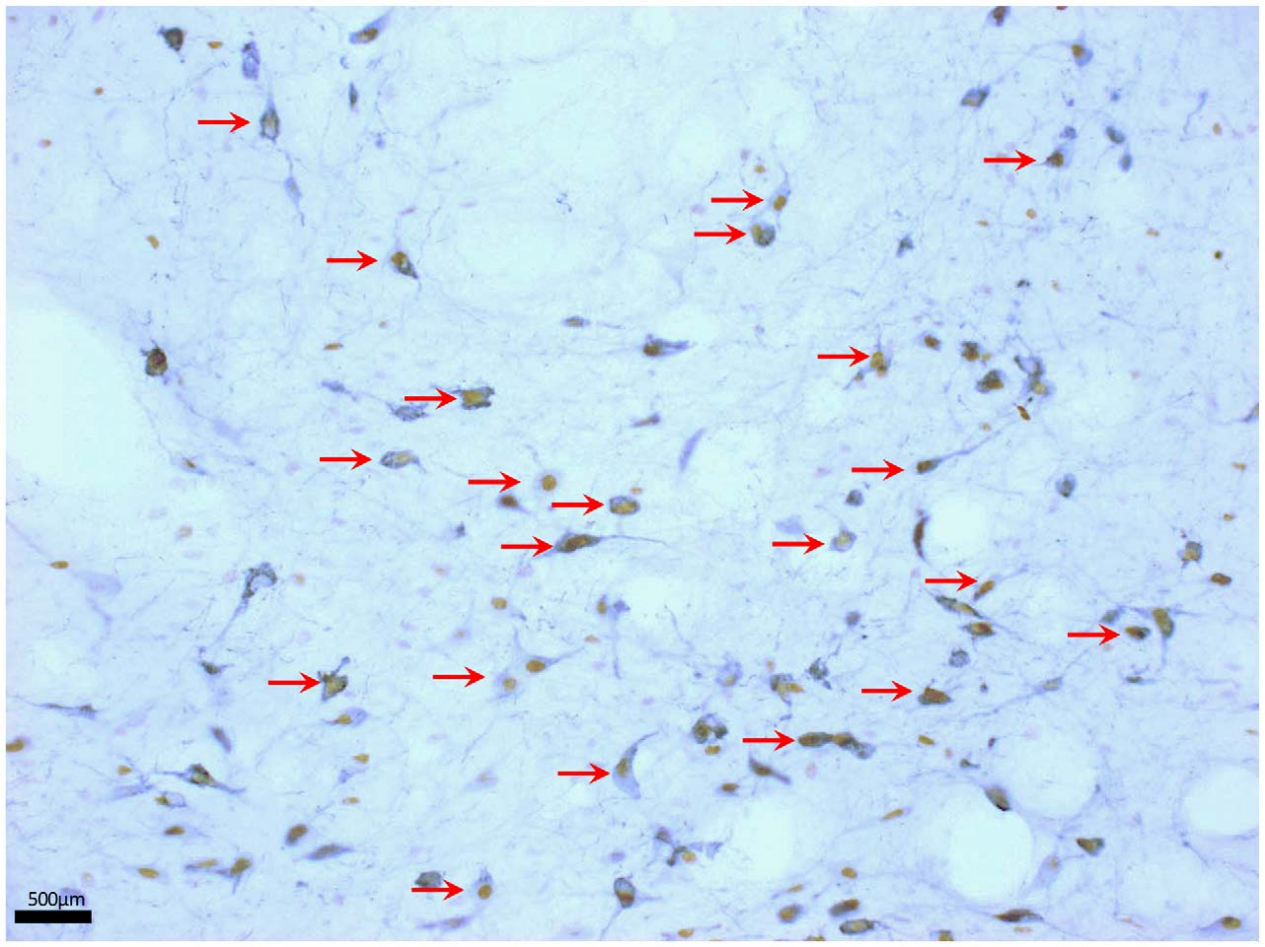
Representative image of orexin cell staining. Orexin cells co-expressing nuclear c-Fos show characteristic brown (c-Fos) nuclear staining and black (orexin) cytoplasmic staining. Examples of co-expressing cells are indicated by red arrows. The fornix lies to the left of the region shown here, just outside the frame of the image.

Two hours after completion of behavioural testing, animals were perfused and brains removed to study the activation of orexin neurons in the lateral hypothalamus. Sections were double immunostained with orexin and c-Fos and the percentage of orexinergic cells expressing c-Fos was used as a measure of activation (Figure 5).

Table 3 shows that there was no significant difference between orexin cell activation in neonatally anesthetized alone (AN), neonatally incised alone (IN) and acute adult incision animals (AN+IN). In other words, a pain history alone or an acute adult incision alone did not activate orexin cells. However, in animals with a pain history plus an adult acute incision (IN+IN) a significant increase in orexin activation occurred. In these re-incised animals, 27.2±4.1% of orexin cells expressed c-Fos, compared to 15.5–18.4% in all other groups (p<0.05, two-way ANOVA). Thus the orexin system is activated following NIH arena testing in animals with a ‘pain history’ when they are re-injured as adults.

**Table 3:** Orexin, c-Fos and co-expressing cell counts in experimental groups 2 hours after NIH arena testing.

Treatment group	Number of animals	Orexin-positive cells per section#	c-Fos-positive cells per section	Number of co-expressing cells per section	% orexin cells co-expressing c-Fos per section
AN	6	42.4±3.7	31.6±6.9	6.5±1.4	16.4±4.5
AN+IN	7	54.6±5.2	28.1±3.2	9.8±1.3	18.4±2.8
IN	7	43.2±4.2	28.2±7.3	7.0±1.5	15.5±2.6
IN+IN	7	48.2±4.9	46.8±14.4	13.7±3.1	27.2±4.1**

# 5 sections per animal were bilaterally counted. All values are expressed as group averages, calculated from averages within animals. Results are mean±SEM. Significant results are highlighted in bold.

*p<0.05, two-way ANOVA with Bonferroni's Multiple Comparison Test.

**Figure 6:**
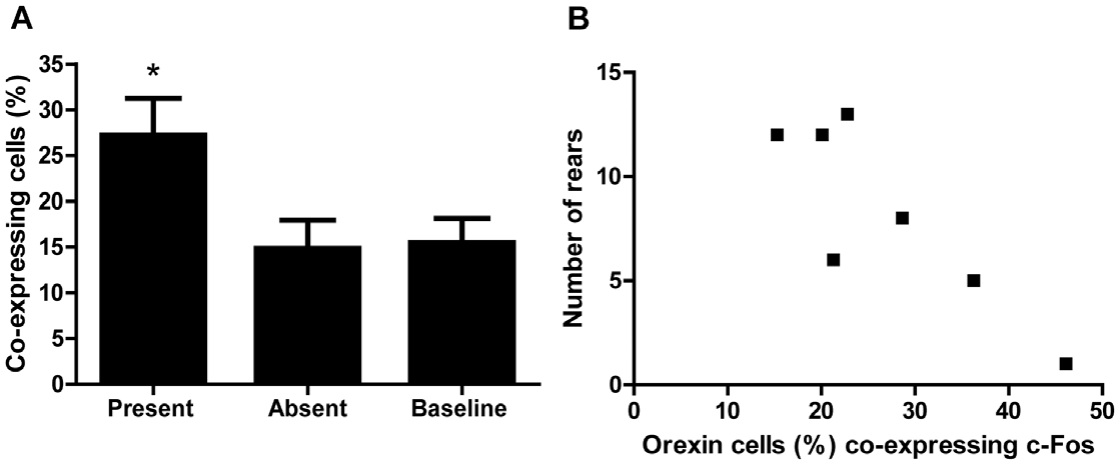
Presence of treats affects orexin activation, which is associated with motivational behaviour. (**a**) The activation of orexin cells in adult re-injured animals with a ‘pain history’ (IN+IN) is dictated by the presence or absence of food treats in the central bowl of the NIH arena. When treats are present in this group, orexin cell activation increases to 27.2±4.1% compared to when treats are absent (14.8±3.1%) and at baseline (15.5±2.6%). ‘Baseline’ data refers to animals with a history of incision but no adult re-injury (IN), tested in the presence of treats. *p<0.05; one-way ANOVA with Bonferroni's post-tests. (**b**) There is a strong correlation within individual animals (r = −0.86, p = 0.013) between the percentage of orexin cells co-expressing c-Fos and the number of rears performed by the IN+IN group during testing in the NIH arena.

To confirm that this increase in activation was specific to the presence of the food treats, separate subsets of animals were tested with or without the treats in the food bowl. In the absence of treats, orexin activation levels returned to baseline levels of ∼15% (p<0.05, one-way ANOVA, Figure 6A).

### 6. Orexin cell activation is correlated with behavioural changes in individual rats with a pain history

The orexin activation was plotted against behavioural outcome measures in individual IN+IN animals. Table 4 and Figure 6B show that orexin cell activation is highly negatively correlated with the number of rears (r = −0.86, p = 0.013), i.e. the number of rears decreases as orexin cell activation increases in this group. This result suggests that activation of the orexin system is related to exploratory behaviours in acutely injured rats with a ‘pain history’.

**Table 4:** Orexin activation is correlated to motivational behaviour in neonatally-incised and re-injured animals.

Treatment group	Rears		Time in central zone (%)	
	Pearson's R	P value	Pearson's R	P value
AN	0.55	0.25 (n.s.)	−0.48	0.44 (n.s.)
AN+IN	−0.16	0.73 (n.s.)	0.32	0.49 (n.s.)
IN	0.69	0.08 (n.s.)	−0.46	0.30 (n.s.)
IN+IN	**−0.86**	**0.0132 (** * **)**	0.02	0.96 (n.s.)

n.s. = not significant.

*p<0.05 (highlighted in bold).

No correlations between the behavioural outcome measures and the percentage of orexin cells co-expressing c-Fos protein were observed in the other groups. This suggests that orexin is only involved in mediating NIH arena behaviour in acutely injured animals when they have a pain history.

## Discussion

These results add to an increasing body of literature showing that the environment to which an organism is exposed during infancy has long-lasting effects upon both sensory and cognitive processing. This study shows that local peripheral tissue injury in infant rats not only alters acute pain sensitivity in adulthood but also causes long-lasting changes in brain function distinct from pain processing - in this case, exploration and motivation. These changes are correlated with activation of orexinergic cells in the lateral hypothalamus.

The results showed that repeated neonatal skin incision did not significantly alter baseline mechanical or thermal withdrawal thresholds in adulthood, although the hyposensitivity reported in other studies may have been present in other areas of the body not tested here [68]–[70]. However, on adult re-incision to these animals, a significant hypersensitivity was observed in the previously injured paw – consistent with previous work [6], [18], [28], [69]. This phenomenon may have parallels with pain hypersensitivity in post-intensive care children exposed to painful stimuli in later life [14], [16], [71].

The mechanism underlying these changes is not known. Early life injury has been reported to alter levels of circulating endogenous opioids in nociceptive-related areas of the brain in the adult [70], which in turn may alter sensitivity of related CNS networks, such as the dopaminergic system. In addition, endogenous opioids activate brainstem descending pathways which, in turn, modulate spinal nociceptive circuits. These brainstem descending control systems are not mature during the first three weeks of a rat's life [72]–[74] and the third week of life is a critical period in the maturation of brainstem opioidergic networks [75]. Thus repeated skin incision over this period may interfere with the normal maturation of central opioidergic circuits and their activation of descending control systems, leading to increased pain sensitivity, altered behavioural responses to future injury, and alterations in alternative opioid-mediated behaviours.

Peripheral nerve damage induced by inflammation or incision during development causes increased axonal sprouting and hyperinnervation of the damaged hindpaw [2], [26], increases in primary afferent nerve fibre innervation of the spinal cord [29], and increased receptive field size of dorsal horn spinal cord cells [3]. Thus changes at the level of the primary afferent input and spinal sensory circuits will also contribute to an increased response to nociceptive input, and altered nocifensive responses in later life [18].

The changes in motivation reported here were measured using a novelty-induced hypophagia (NIH) arena, where the anxiety of moving into the centre of an open field is balanced by the reward of a sweet food treat. This behavioural test has not previously been used in pain models, but may be a useful model for research on the impact of pain on cognition, and has been validated for anxiolytic and antidepressant drug effects [46]. It requires little or no training, can score a number of outcome measures such as rearing, locomotion, exploration and consumption, in addition to classic ‘emotionality’ measures such as defecation and urination [76]. Furthermore, it is an ethologically relevant test that mimics real-world experiences, making it more valid to the natural behaviour of a rodent than complex operant tests such as the five-choice serial reaction time task [77]. However, this task produces an inherent conflict between the approach of food versus the anxiety this induces, and these are difficult to discriminate. We included outcome measures of ‘emotionality’ such as defecation to separate out the concepts, but it would be interesting in future to validate the anxiogenic effects using a classical anxiety paradigm such as the elevated plus maze.

Our results show that neonatal exposure to either surgery or anesthesia (IN or AN) does not, in itself, influence adult exploratory behaviours or approach to a food reward. This may be because these animals were tested in the absence of a further painful stimulus: neonatal injury ‘primes’ animals to respond to a new noxious insult in adulthood to a greater extent than neonatally uninjured animals [6], [18], [27], [31]. We suggest that this is why our neonatally incised animals did not show alterations in their motivational and exploratory behaviours in the ‘baseline’ state - it is the presence of a new insult that ‘unmasks’ altered behaviour, when brain systems that were altered during development are recruited. Interestingly, there were an unexpectedly high proportion of these rats that did not eat any treats whilst in the arena, despite spending as long as controls in the vicinity of these treats. One possibility is that this could result from an underlying anhedonia (lack of pleasure) for food reward in these males. That the IN group showed no differences in orexin cell activation to the other groups would suggest that this anhedonia may be modulated by signalling mechanisms discrete from orexin i.e. serotonergic signalling. Further studies would be needed that specifically address anhedonic behaviour i.e. the sucrose preference test [78], and it's associated neurotransmitter systems, to confirm this hypothesis.

An acute adult incision (AN+IN) caused significant changes in behaviour - a reduction in frequency of rearing, and an increase in time spent in the centre of the arena (in close proximity to the bowl of food treats). The decrease in rearing was not due to incision alone, as when treats were removed (Fig. 4C) animals injured 48 hours previously reared as often as control (non re-injured) animals, suggesting that rearing in this context is an index of exploration. The fact that these animals spent more time in the centre of the arena, a bright and anxiogenic environment, suggests that whilst the motivation to explore may be decreased (as shown by decreased rearing in the arena), the motivation to approach the food treats is increased (as shown by the increase in time spent in the centre) [79]. The lack of change in rate of eating or overall amount of consumption could reflect the inherent tendencies of rodents to take longer to eat familiar foods in novel environments, termed novelty-induced suppression of feeding [42], [44], [80]. Importantly however, testing animals in the absence of treats caused the time spent in the central zone to return to baseline levels, showing that the treats were indeed driving approach behaviours. These data are consistent with literature showing that an acute adult injury can affect cognitive processing, for example attention, recognition of novel objects, and place preference [81]–[83].

Acute injury in rats with a neonatal ‘pain history’ (IN+IN) also caused significant shifts in motivational behaviour in the NIH arena that were not different to those of the acutely-injured alone animals, although interestingly these animals defecated more than others, suggesting that the NIH arena is inducing anxiety in this group, but that they approach the food reward regardless. One possibility for the lack of differences between AN+IN and IN+IN on other outcome measures may be that this behavioural measure lacked the sensitivity to detect any differences, or that a ceiling effect had been reached. However, there was a significant increase in orexin cell signalling in the lateral hypothalamus after NIH arena testing of acutely injured animals with a neonatal ‘pain history’, which was not seen in acutely-injured alone animals. This suggests that, despite the similarities in the behavioural outcomes measured here, the neurobiological mechanisms mediating the behaviours in these two groups are different. As orexin activation and correlations with behaviour were specifically seen in IN+IN animals, not AN+IN animals, we postulate that orexin signalling is mediating the behaviour seen in the IN+IN group. Furthermore, if there is an underlying anhedonia in neonatally injured alone animals, that there was no reduction in the number of animals eating treats in the IN+IN group supports the idea that a new incision in adulthood ‘unmasks’ motivational behaviours that are being driven by the orexin system.

The role of orexin peptide release from lateral hypothalamic orexin cells has been extensively investigated in terms of reward processing, and orexin signalling has been shown to be crucial for cue-drug association learning [51], [84], morphine-induced conditioned place preference [85], abstinence [86], and reinstatement [87], [88]. Orexins excite dopamine cells in the ventral tegmental area (VTA) [54] and increase glutamate-dependent LTP of VTA neurons in the same manner as addictive drugs such as cocaine [57], [58], [89] therefore directly affecting dopaminergically-mediated systems. Furthermore, it is likely that orexin action on mesolimbic dopamine circuitry serves to promote effort to obtain highly salient natural or drug rewards [53], placing orexin signalling in a crucial position to influence the dopaminergic system. We hypothesised that orexin cell activation, due to its direct interaction with dopaminergic pathways and mediation of dopaminergic signalling, would be a biomarker of alterations in dopaminergic tone caused by repeated neonatal injury affecting the balance of the developing system.

The fact that increases in orexin activation failed to occur when food treats were not present suggests that it is the presence of food treats that triggers orexin, and hence dopamine, activation. Importantly, this activation was correlated within subjects to rearing behaviour, strongly suggesting that rearing behaviour in these animals is dopaminergically mediated. We did not see any correlation of behaviour with orexin activation in animals that received only a single acute adult injury, suggesting that the behaviours seen after acute injury are not dopaminergically mediated via the orexin system. This is some of the first data, to our knowledge, where orexin activation is seen in a non-operant reward task and it would be interesting to test whether orexin antagonists block the behaviours observed, which would confirm a direct link between orexin signalling and NIH arena behaviour. Future studies should also address the speculative link to dopaminergic signalling mentioned above i.e. by delivery of dopamine receptor agonists and antagonists whilst testing in the NIH arena.

Intriguingly, orexin system activation was negatively correlated with rearing behaviour i.e. the fewer rears the animal performed, the more orexin activation was seen. Rearing is often described as an exploratory behaviour [90] – decreased rearing after an adult injury suggests that exploratory drive was decreased in these animals. We argue that the orexin system was driving approach behaviours towards the central food bowl instead, meaning the animal was spending more time in proximity to the treats than exploring in the arena. That we did not see a correlation between time in CZ and orexin activation is therefore puzzling. At this point, that a link was seen between orexin signalling and behaviour suggests further work into the effects of early pain on reward system development is merited.

These findings are clinically important as they show that neonatal pain experience can have long-term effects, not only upon adult pain sensitivity but also upon the sensitivity of central reward pathways to an acute injury. Previous research has shown that length of stay on the neonatal intensive care unit (NICU) correlates to changes in pain processing in older life [16] and longitudinal studies following the progress of post-NICU children are now beginning to show that NICU experience can lead to increased risk of behavioural problems, lower IQ, and learning impairments [91]–[93]. Understanding the phenotypic changes that can occur after early life intensive care experience could help reduce the social and economic costs associated with negative outcomes.

Females were not included in these analyses as hormonal fluctuations were not monitored, which is a limitation of this study. The literature has shown that hormonal levels affect nociception [31], [94], [95] and it would be interesting to investigate the effects of the menstrual cycle on behaviour in the NIH arena. Another potential confound in the interpretation of these results is the effect of litter size, which ranged from 6–16 pups. Smaller litters gain weight faster and open their eyes earlier than larger litters [96]–[98], suggesting that incision at P3, 10 and 17 may be having differential effects on different litter sizes. However, all animals were at least P22 when weaning commenced, allowing for animals from larger litters to reach comparable weights and developmental milestones as those from smaller litters. Furthermore, the critical aspect of the experiment was the repetition of skin incision over the pre-weaning period, and therefore all animals were subject to the same stimulation within comparable developmental windows.

In conclusion, these experiments show that an acute adult skin incision causes behavioural changes towards a palatable food treat, regardless of the ‘pain history’ of the animal. However, animals with a history of incision show an increase in activation of the lateral hypothalamic orexin system when exposed to food reward, which correlates with exploratory behaviours displayed in the NIH arena. Early life experience is known to affect nociceptive processing and stress responsivity, but these data add to research from the pain field showing that early life painful experience can impact upon modalities *outside* of nociception [27], [99]. In addition, these data are some of the first to explicitly show that acute adult injury shifts reward and exploration behaviours. The existence of a critical period of nociceptive development, plus the extensive overlap between reward and pain processing, suggest that interference with one modality (i.e. nociception) during this critical period can impact upon the development of the other (i.e. reward pathways). Our results support this hypothesis, and warrant more research into the long-term effects of neonatal pain exposure upon non-nociceptive modalities, which would help inform clinical practice in its treatment of young children.
